# Natural therapeutics and traditional formulas targeting macrophage polarization in the fibrotic niche of idiopathic pulmonary fibrosis

**DOI:** 10.3389/fimmu.2026.1860332

**Published:** 2026-06-30

**Authors:** Lei Han, Yifeng Sun, Yun Wang, Qian Wang, Xi Zhang, Huan He, Jia Wang

**Affiliations:** 1 Hongqi Hospital Affiliated to Mudanjiang Medical University, Mudanjiang, China; 2 Xuyi County People’s Hospital, Huai’an, Jiangsu, China; 3 Mudanjiang Medical University, Mudanjiang, China

**Keywords:** fibrotic niche, idiopathic pulmonary fibrosis, macrophage polarization, mechanotransduction, natural therapeutics

## Abstract

Idiopathic pulmonary fibrosis (IPF) is a progressive interstitial lung disease driven by a self-perpetuating “fibrotic niche,” where monocyte-derived alveolar macrophages (Mo-AMs) regulate disease progression. Conventional single-target antifibrotics show limited efficacy in reversing established fibrosis and frequently cause dose-limiting toxicities, prompting the investigation of natural product-based therapeutics as multi-targeted investigational alternatives. This narrative review examines the mechanisms through which purified phytochemicals and multicomponent botanical formulas modulate the pulmonary fibrosis (PF) immune microenvironment. Moving beyond the classical M1/M2 dichotomy, we outline the spatiotemporal heterogeneity of macrophages and their bidirectional crosstalk with myofibroblasts. We summarize preclinical and experimental pharmacological interventions targeting macrophage reprogramming networks, detailing how natural monomers exhibit targeted immunomodulation, including immunometabolic shifts (e.g., covalent GAPDH inhibition to suppress glycolysis) and mechanotransduction blockade (e.g., Piezo1 inhibition by specific terpenoids). Concurrently, quality-controlled botanical formulas demonstrate potential synergistic effects in modulating microenvironmental homeostasis. Natural product-based therapeutics provide a promising investigational approach for modulating the fibrotic niche. To address the translational gap in this field, future research requires a methodological shift from theoretical network pharmacology to empirical validation using raw metabolomics and spatial transcriptomics, integrated with macrophage-targeted nano-delivery systems and rigorous chemical fingerprinting.

## 1 Introduction

Idiopathic pulmonary fibrosis (IPF) is a progressive and fatal interstitial lung disease. It is characterized by aberrant extracellular matrix (ECM) deposition, structural distortion, and a continuous decline in pulmonary function. IPF presents significant clinical and socioeconomic challenges. Recent data show crude incidence rates ranging from 14.5 to 26.1 per 100,000 person-years and a prevalence up to 88.9 per 100,000 persons. These rates increase exponentially with age (1). The prognosis is poor; the median survival time is 3 to 5 years from diagnosis, and the five-year mortality rate reaches 69%. The standardized mortality ratio is 4.66 compared to the general population, which is comparable to several aggressive malignancies (1, 2). Furthermore, the unmet therapeutic needs in IPF extend to acute exacerbations (AE-IPF), where treatment remains uncertain. In this context, recent evidence syntheses highlight the complex role of corticosteroid use in managing these severe respiratory deteriorations (3, 4).

Currently, two FDA-approved antifibrotic agents, nintedanib and pirfenidone, remain the evidence-based standard of care for IPF. Clinical trials (such as INBUILD and ASCEND) show these drugs reduce the annual decline in forced vital capacity (FVC) by approximately 100 to 110 mL compared to placebo. However, these treatments do not stop disease progression (5, 6). Neither agent can reverse existing fibrotic remodeling or resolve honeycomb cysts. Additionally, they do not provide a proven long-term survival benefit (6, 7).

Furthermore, the clinical use of these agents is limited by dose-dependent toxicities and high treatment costs (exceeding $100,000 annually per patient) (5). Nintedanib is associated with gastrointestinal and hepatic toxicity. In clinical studies, diarrhea occurs in approximately 67% of patients, and elevated aminotransferase levels require routine monthly monitoring (6). Pirfenidone frequently causes photosensitivity, anorexia, and dyspepsia (7). These adverse events lead to dose reductions, treatment interruptions, or discontinuation in up to 20% of patients (5, 6). The limited efficacy and high incidence of adverse effects indicate a need for new, well-tolerated, and multi-targeted therapeutic strategies to treat pulmonary fibrosis. Given these limitations, recent literature has increasingly focused on emerging pharmacological approaches to halt or reverse pulmonary fibrosis (8, 9).

Over the past decade, the understanding of IPF pathophysiology has shifted. Instead of focusing solely on fibroblasts or classical inflammation, research now emphasizes a self-perpetuating “fibrotic niche” (10). This microenvironment is a localized system that includes injured alveolar epithelium, activated myofibroblasts, immune cells, and a stiffened ECM (11). This niche functions through mechanical feed-forward loops. Normal alveolar tissue has a physiological stiffness of 1–5 kPa. In contrast, fibrotic regions with excessive collagen cross-linking reach a stiffness of 10–20 kPa (11). This mechanical change activates mechanotransduction pathways via ion channels like Piezo1, integrins, and the YAP/TAZ signaling axis (12). These pathways drive the fibroblast-to-myofibroblast transition (FMT). This process makes ECM remodeling self-sustaining and independent of the initial inflammatory trigger (11, 12).

Within this microenvironment, macrophages are primary regulators of local immune responses (13). Beyond the traditional M1 (pro-inflammatory) and M2 (pro-fibrotic) classification, single-cell RNA sequencing (scRNA-seq) has shown significant macrophage heterogeneity and plasticity during IPF progression (14). Specifically, Mo-AMs recruited to the fibrotic niche undergo metabolic and phenotypic changes. These changes are driven by microenvironmental factors like iron overload and lipid accumulation (15). Specific pathogenic subpopulations, such as SPP1-high (osteopontin-expressing) macrophages, localize mainly within fibroblastic foci (16). These macrophages interact with myofibroblasts through paracrine mediators (e.g., TGF-β, PDGF, and CCL18) and direct physical contact, promoting fibrogenesis (15, 16). Therefore, targeting this macrophage-mediated microenvironment is a potential therapeutic approach.

Because the fibrotic niche involves complex cellular networks and mechanical feedback, single-pathway interventions are often insufficient. Inhibiting one cytokine typically activates compensatory pathways. Consequently, natural therapeutics, including botanical monomers and standardized TCM formulas, are being investigated as multi-targeted investigational approaches (17, 18). These therapeutics can modulate multiple pathways simultaneously. They have been shown in preclinical models to suppress profibrotic macrophage polarization, inhibit mechanotransduction signaling, and reduce oxidative stress, generally demonstrating fewer adverse effects than synthetic antifibrotics (19, 20).

This manuscript is structured as a narrative review. The primary objective is to synthesize current preclinical and clinical evidence regarding the mechanisms through which natural products and traditional botanical formulas modulate macrophage polarization in the IPF fibrotic niche. Literature was surveyed across major biomedical databases focusing on studies that investigate macrophage heterogeneity, microenvironmental crosstalk, and the pharmacological mechanisms of natural therapeutics. We categorize current therapeutic evidence into two main areas (1): natural monomers and (2) quality-controlled TCM formulas. By examining their effects on macrophage immunometabolism, phenotypic transition, and microenvironmental crosstalk, this review aims to provide a comprehensive conceptual framework rather than a quantitative systematic meta-analysis, thereby contextualizing these natural interventions as investigational approaches within the broader therapeutic landscape of IPF.

## 2 The fibrotic niche and macrophage heterogeneity

### 2.1 Transcending the M1/M2 paradigm: ontological origins and scRNA-seq signatures

Advances in scRNA-seq, spatial transcriptomics, and high-dimensional mass cytometry show that the traditional M1 (pro-inflammatory) and M2 (pro-fibrotic) macrophage classification is inadequate for progressive interstitial lung disease (14, 21). High-resolution multi-omics indicate that the fibrotic niche exhibits spatiotemporal heterogeneity. This heterogeneity is primarily determined by the ontological origins and developmental trajectories of different macrophage populations in the lung.

Under homeostatic conditions, tissue-resident alveolar macrophages (TR-AMs) populate the alveolar space. These cells originate from embryonic progenitors (the yolk sac and fetal liver) and self-renew locally without adult hematopoiesis. They are transcriptionally characterized by the expression of MARCO, SIGLEC-F (in mice) or SIGLEC-8 (in humans), and PPARG (22). TR-AMs require specific microRNA networks (e.g., let-7a, miR-155, and miR-125) for postnatal maintenance. Following severe or repetitive pulmonary injury, this resident population decreases. Multiplatform analyses show that TR-AMs do not possess significant profibrotic capacity. Genetic ablation of TR-AMs in bleomycin-induced murine models does not halt or attenuate fibrogenesis, indicating they are not primary drivers of the fibrotic process (22).

In contrast, Mo-AMs repopulate the injured areas. They are recruited from circulating Ly6Chi (mice) or CD14+ (humans) monocytes via chemokine gradients, such as CCL2. These cells differentiate within the alveolar and interstitial spaces (22). Mo-AMs require M-CSF/M-CSFR (macrophage colony-stimulating factor receptor) signaling for survival and are primary drivers of fibrosis. Lineage-tracing studies show that Mo-AMs are necessary for disease progression. Pharmacological blockade or genetic depletion of Mo-AMs abrogates experimentally induced pulmonary fibrosis, demonstrating their specific role in fibrogenesis (15).

Within the Mo-AM population, scRNA-seq identifies several specialized subpopulations. A major subset is the SPP1-high (osteopontin-expressing) macrophage population. This group demonstrates local proliferation and upregulates disease-associated markers (SPP1, MERTK, TREM2, CD9, GPNMB). These cells also show a lipid metabolism signature (FABP5, APOE, APOC1, LPL) and express matrix remodeling enzymes (MMP12, MMP14, CTSK) (16, 23). Multiomic single-nucleus ATAC/RNA-sequencing shows that AP-1 (FOS/JUN family) and bHLH-ZIP transcription factors drive the differentiation of this phenotype by increasing chromatin accessibility at fibrotic gene enhancers (23). Spatial transcriptomics and single-molecule fluorescence *in situ* hybridization confirm that SPP1-high macrophages localize to fibroblastic foci. They interface directly with ACTA2+ myofibroblasts, forming a key structural component of the fibrotic niche (16).

Trajectory analyses reveal developmental plasticity within the macrophage populations. Transitional cells, such as CX3CR1+SiglecF+ macrophages, represent an intermediate differentiation state co-expressing monocyte and alveolar markers. These cells exhibit strong profibrotic capacity before acquiring mature alveolar characteristics (24). Additionally, metabolically distinct subpopulations, such as PLA2G7-expressing macrophages, are present. Elevated in human IPF and murine models, these macrophages promote the FMT through a lipid signaling axis. PLA2G7 generates lysophosphatidylcholine (LPC), which autotaxin converts into lysophosphatidic acid (LPA). LPA binds to the LPA2 receptor on fibroblasts, inducing α-SMA expression (25). This subpopulation structure indicates that fibrotic progression depends on the specific transcriptomic and metabolic states of Mo-AMs rather than general inflammation.

### 2.2 Microenvironmental crosstalk

The fibrotic niche functions as a spatially organized system. Heterogeneous Mo-AMs, injured alveolar type II (AT2) epithelial cells, and myofibroblasts interact through paracrine and mechanical signaling (10, 26). This signaling network forms a feedback loop, contributing to the continued progression of IPF after the initial injury resolves.

Mo-AMs promote the FMT by secreting paracrine mediators. A primary mediator is TGF-β, a key fibrogenic cytokine. Macrophage-derived TGF-β activates canonical Smad2/3 signaling pathways, inducing α-SMA expression, stress fiber formation, and ECM protein synthesis (including collagen I/III and fibronectin) (27). Localized iron overload in the fibrotic niche increases macrophage-derived TGF-β production by triggering intracellular lipid peroxidation (28). Mo-AMs also secrete platelet-derived growth factor subunit A (PDGF-A), which acts as a mitogen and chemoattractant to promote fibroblast proliferation and migration (28). Furthermore, macrophage-derived cytokines, such as IL-1β, upregulate PDGF-Rα receptors on fibroblasts, increasing their response to proliferative signals.

Macrophage-fibroblast signaling also involves the chemokine CCL18. Profibrotic Mo-AMs secrete CCL18, which stimulates fibroblast collagen production through Sp1-dependent transcriptional signaling, independent of autocrine TGF-β (29). The synthesized collagen monomers subsequently activate adjacent macrophages via the CD204 (scavenger receptor class A) receptor. Collagen-CD204 binding activates phospho-Akt pathways, reinforcing the profibrotic macrophage phenotype and promoting further matrix deposition (29, 30). Connective Tissue Growth Factor (CTGF/CCN2), expressed by AT2 cells and fibroblasts, enhances TGF-β-mediated responses. This accelerates the epithelial-mesenchymal transition (EMT) and contributes to fibrotic structural changes (31, 32).

Cellular activation is additionally reinforced by signals from the injured epithelium. Dysfunctional AT2 cells undergoing apoptosis or exhibiting a Senescence-Associated Secretory Phenotype (SASP) secrete damage-associated molecular patterns (DAMPs), such as HMGB1 and extracellular ATP (33, 34). Driven by p21 and p16 pathways, these senescent AT2 cells secrete IL-6, PAI-1, and macrophage migration inhibitory factor (MIF). MIF acts through CD74 signaling to recruit circulating monocytes to the injury site (34). Fibrosis-associated AT2 cells also secrete Sonic hedgehog (Shh). Shh signaling via the Shh/Gli pathway promotes osteopontin secretion in recruited macrophages, maintaining their profibrotic state (33).

**Figure 1:**
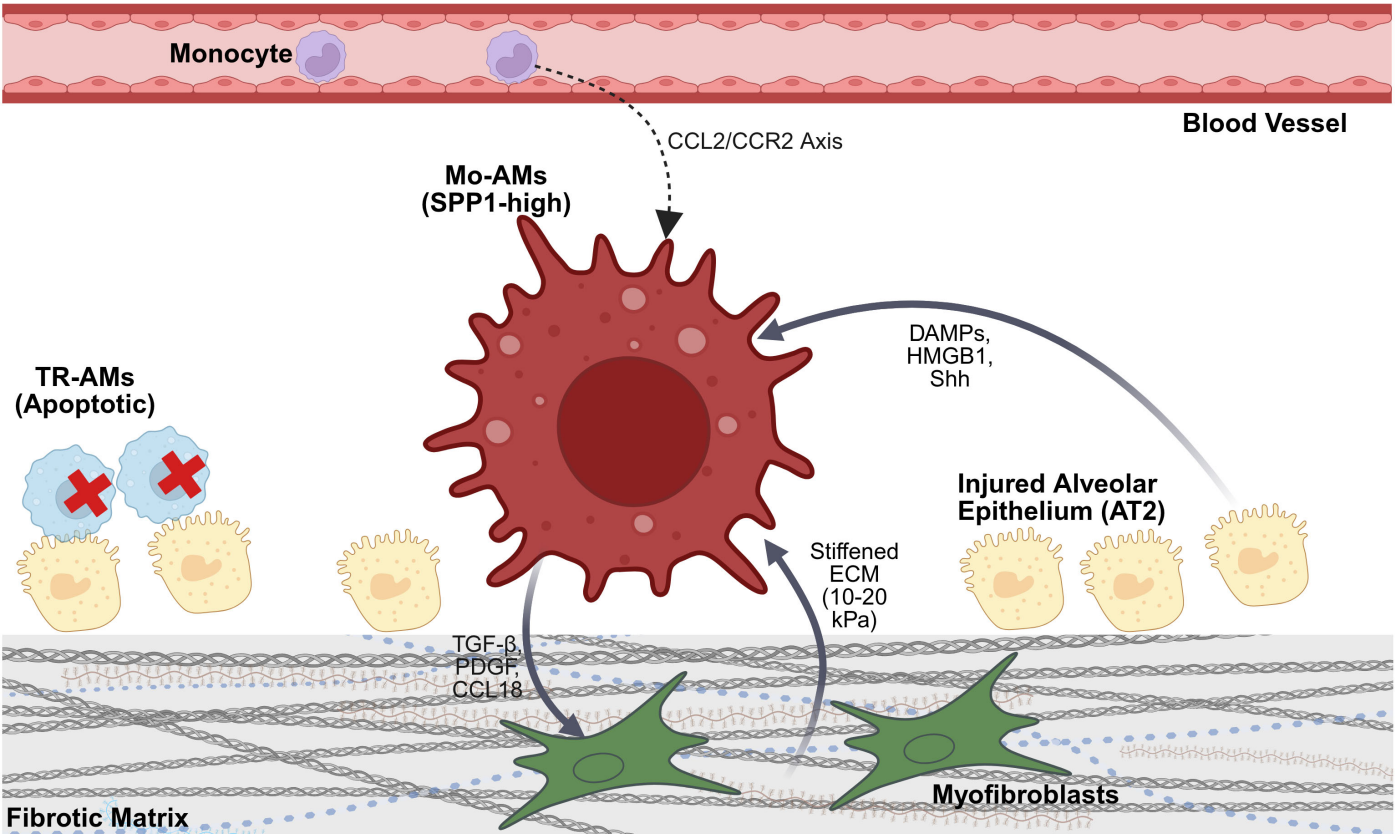
Crosstalk between macrophage ontogeny and the fibrotic niche in IPF (Created with BioRender.com.). During the pathogenesis of IPF, TR-AMs undergo apoptosis following alveolar epithelial cell (AT2) injury. Concurrently, circulating monocytes are recruited via the CCL2/CCR2 axis and differentiate into highly pro-fibrotic monocyte-derived alveolar macrophages (Mo-AMs, characterized by high SPP1 expression). Within the fibrotic niche, a vicious cycle of paracrine signaling emerges: injured AT2 cells secrete DAMPs, HMGB1, and Shh to persistently activate Mo-AMs. Activated Mo-AMs subsequently release pro-fibrotic mediators, including TGF-β, PDGF, and CCL18, driving the activation and proliferation of myofibroblasts. The progressive deposition and stiffening of the ECM establish a rigid microenvironment (10–20 kPa), which provides aberrant mechanotransduction feedback to Mo-AMs, further perpetuating the fibrotic cascade. (The cellular dynamics depicted here are synthesized primarily from preclinical murine models and *in vitro* human cell data, and represent a proposed model of the fibrotic niche.).

As the ECM stiffens due to collagen deposition and cross-linking, mechanotransduction affects cell behavior (35). Macrophage TRPV4 channels respond to substrate stiffness in the 10–20 kPa range. In response to this stiffness, TRPV4 initiates actinomyosin cytoskeleton assembly. This generates cellular forces that activate latent TGF-β stored in the ECM (27). Integrins and mechanosensitive ion channels (like Piezo1) on macrophages and fibroblasts detect mechanical stress, activating focal adhesion kinase (FAK) and the YAP/TAZ signaling axis (35). This mechanotransduction pathway allows Mo-AMs to remain activated by the stiffened microenvironment, independent of ongoing immune or cytokine stimulation. This mechanical feedback loop contributes to the self-sustaining nature of the fibrotic niche (**Figure 1**).

## 3 Mechanisms of macrophage reprogramming

Macrophage reprogramming within the fibrotic niche is regulated by classical signaling cascades, bioenergetic shifts, and physical mechanosensing. These mechanisms interact to maintain Mo-AMs in a profibrotic state. Therefore, targeting these molecular and biomechanical pathways is a primary objective for developing natural therapeutics.

### 3.1 Classical inflammatory and profibrotic signaling cascades

The polarization of macrophages in IPF involves three classical signaling cascades: NF-κB, PI3K/Akt, and TGF-β/Smad. Evidence indicates that these pathways form interconnected networks that can be modulated by natural therapeutics.

The NF-κB and PI3K/Akt Axes: Canonical NF-κB (p65) signaling drives early pro-inflammatory macrophage activation via Toll-like receptor 4 (TLR4). Its sustained activation is triggered by the fibrotic ECM, linking inflammation to matrix remodeling (13, 36). Conversely, the p50 subunit of NF-κB promotes a profibrotic, matrix-producing phenotype (37). The PI3K/Akt pathway regulates macrophage survival and profibrotic activation. The AKT2 isoform phosphorylates and inactivates the transcription factor FoxO3a, leading to increased IL-13 and TGF-β1 production (38). Additionally, Akt1 induces mitophagy, which confers apoptosis resistance to these macrophages (39).

Natural therapeutics can suppress multiple pathways simultaneously. For example, the multi-herb TCM preparation Fuzheng Huayu suppresses p65 nuclear translocation, reducing early macrophage-driven inflammation (36). Additionally, scRNA-seq and molecular docking show that the flavonoid baicalein directly binds to the catalytic and regulatory subunits of PI3K (40). Similarly, Icariside II downregulates profibrotic markers (CD206, Arg-1) by inhibiting the PI3K/Akt and β-catenin pathways, decreasing profibrotic macrophage infiltration (41).

The TGF-β/Smad Axis: The progression from macrophage activation to FMT depends on TGF-β/Smad signaling. Following receptor activation, phosphorylated Smad2/3 complexes translocate to the nucleus to promote ECM synthesis (13). The Renshen Pingfei Formula (containing 78 identified active components) suppresses Smad2/3 phosphorylation and reduces profibrotic surface markers on macrophages in alveolar and interstitial compartments (19). Botanical extracts such as Schisandra upregulate Smad7, an inhibitory protein that blocks Smad2/3 complex formation (42).

### 3.2 Immunometabolic reprogramming

Macrophages in the fibrotic niche undergo immunometabolic reprogramming. Unlike classically defined *in vitro* alternative macrophages that rely primarily on OXPHOS, Mo-AMs exhibit glycolytic dependence to maintain their profibrotic phenotype (43).

The HIF-1α and Glycolytic Feedback Loop: Fibrotic macrophages show elevated extracellular acidification rates (ECAR) due to the upregulation of glycolytic enzymes, such as hexokinase 2 (HK2) and PFKFB3 (43). This metabolic shift is regulated by Hypoxia-Inducible Factor-1α (HIF-1α). Increased glycolysis produces excess intracellular succinate. Succinate inhibits prolyl hydroxylases (PHDs), preventing HIF-1α degradation and establishing a metabolic feedback loop (44, 45). Additionally, HIF-1α promotes profibrotic macrophage activation by mediating STAT6 arginine methylation via PRMT1 (44). Furthermore, IPF macrophages show reduced levels of itaconate, an endogenous metabolite. The suppression of the ACOD1 gene (responsible for itaconate synthesis) limits the inhibition of succinate dehydrogenase (SDH) and the NLRP3 inflammasome, contributing to fibrogenesis (46).

Natural Products as Metabolic Modulators: Botanical compounds can modulate macrophage metabolism. A 2025 study using ABPP showed that baicalein acts as a targeted covalent inhibitor. It modifies the catalytic cysteine residue (Cys150) of GAPDH, reducing glycolytic flux and promoting a metabolic shift toward OXPHOS in macrophages (47). Polygonum cuspidatum extracts inhibit the HIF-1α/STAT3 axis, downregulating HK2 and PFKFB3 to reduce glycolytic dependence (48). Regarding lipid metabolism, the SPP1-high macrophage subset expresses lipid handling genes (FABP5, APOE). The flavonoid isoliquiritigenin inhibits aldose reductase, suppressing fatty acid synthesis and reducing profibrotic polarization via ERK/MYC pathway modulation (49). Similarly, berberine activates AMPK, which inhibits the mTORC1/HIF-1α pathway and reduces glycolysis (50).

### 3.3 Mechanotransduction

The biomechanical microenvironment contributes to the progression of fibrosis. As the ECM stiffens to levels of 10–20 kPa, macrophages utilize mechanosensors to convert physical tension into profibrotic biochemical signals (11, 35).

Piezo1 and Mechanosensing: Piezo1, a mechanically activated non-selective cation channel, acts as a stiffness sensor in macrophages (12, 51). Increased membrane tension opens the Piezo1 pore, leading to intracellular Ca^2+^ influx. This calcium influx promotes actin polymerization, which increases macrophage sensitivity to ECM stiffness (51). Piezo1 activation also induces the nuclear localization of YAP/TAZ. This upregulates CCL2 secretion via Notch signaling, facilitating the recruitment of circulating monocytes into the fibrotic areas (12, 52).

Integrins, FAK, and TRPV4: Alongside Piezo1, integrins (e.g., α4β1, αMβ2) connect the stiffened ECM to the cellular cytoskeleton (53). Integrin clustering activates FAK, which induces the ERK-dependent secretion of MCP-1 and promotes macrophage chemotaxis (54). Additionally, the mechanosensitive channel TRPV4 responds to substrate rigidity by initiating actinomyosin assembly. This generates cellular traction forces that mechanically activate latent TGF-β stored in the ECM (27).

Pharmacological Disruption of Mechanosensory Loops: Targeting mechanotransduction pathways is a potential therapeutic strategy for IPF. Research indicates that paeoniflorin, a monoterpene glycoside, acts as a direct inhibitor of Piezo1. Molecular docking and surface plasmon resonance demonstrate that paeoniflorin competitively binds to Piezo1, reducing Yoda1-induced Ca2+ influx and inhibiting the YAP/TAZ signaling pathway (55). The approved antifibrotic agent pirfenidone also functions as a mechanotransduction inhibitor. It suppresses integrin αMβ2 and ROCK2, which prevents the RhoA/ROCK-mediated cytoskeletal remodeling required for macrophage mechanical activation (56, 57). Additionally, monoclonal antibodies against integrins (αM, αMβ2) have shown *in vivo* efficacy in reducing the pro-fibrotic secretome (58). These compounds and agents reduce macrophage sensitivity to ECM stiffness, inhibiting the mechanical feedback loop in fibrosis.

## 4 Natural therapeutics: precision modulators of macrophage polarization

The use of natural therapeutics in pulmonary fibrosis has transitioned from empirical crude extracts to the application of precision pharmacology. Structural classes of natural monomers, including flavonoids, polyphenols, alkaloids, and terpenoids, demonstrate specific structure-activity relationships (SAR). Instead of acting solely as non-specific antioxidants, their distinct pharmacophores (e.g., quinone methides, pinane skeletons, and quaternary nitrogens) facilitate targeted molecular interactions. These interactions include irreversible covalent conjugations and allosteric modulation. These structurally defined agents can modulate the metabolic and mechanosensory pathways involved in profibrotic macrophage polarization. The precise molecular targets, binding mechanisms, and resulting phenotypic changes of these representative natural monomers are summarized in **Table 1**.

**Table 1:** Targeted modulators of macrophage polarization: structural classes, molecular targets, phenotypic outcomes, and evidence stratification in fibrosis.

Compound class	Natural monomer	Primary molecular target(s)	Mechanism of target engagement	Macrophage phenotypic shift/cellular outcome	Evidence level & model	Ref.
Flavonoids	Baicalein	GAPDH (Cys150), PI3K, KEAP1	Covalent modification via quinone methide (ABPP confirmed); ATP-competitive inhibition; PPI disruption	Severe glycolysis suppression; Attenuates profibrotic Mo-AM state; Disrupts fibroblast crosstalk	Preclinical (In vitro & In vivo BLM IPF model)	(55, 57–59)
	Quercetin	NF-κB p65, FSTL1	Phosphorylation inhibition; Transcriptional suppression	↓ NF-κB activation; Suppresses early pro-inflammatory activation	Preclinical (In vivo BLM IPF model)	(60)
Polyphenols	Salvianolic acid B	Mincle-Syk-PKCδ, SP1, TGF-β/Smad	Kinase cascade inhibition; DNA binding inhibition	Attenuates profibrotic macrophage activation; ↓ Macrophage senescence (SASP)	Preclinical (In vitro & In vivo IPF models)	(47)
	Curcumin	PPARγ, NF-κB	Transcriptional activation (PPARγ/RXR-α heterodimer); Nuclear translocation blockade	Inhibits TGF-β1-dependent fibroblast differentiation via PPARγ; ↓ Paracrine profibrotic signaling	Preclinical (In vitro lung fibroblasts)	(61)
	Resveratrol	SIRT1/SIRT3, AMPK	Allosteric activation; Phosphorylation activation	Metabolic bioenergetic shift; ↑ Autophagy; ↓ Oxidative stress-driven inflammation	Speculative in IPF (In vitro non-alveolar macrophage model)	(62)
Alkaloids	Berberine	AMPK, mTORC1, HIF-1α	Direct binding to AMPK regulatory subunit; ↑ AMP/ATP ratio	Glycolysis→OXPHOS shift; Attenuates profibrotic state; ↓ Inflammatory cytokines	Speculative in IPF (In vivo arthritis model)	(40)
	Tetrandrine	L-type Ca2+ channels, IκBα	Calcium channel blockade; Degradation inhibition	↓ Calcium-dependent mechanosensing; ↓ IκBα degradation	Preclinical (In vitro & In vivo models)	(40)
Terpenoids	Paeoniflorin	Piezo1, HIF-1α	Direct binding to channel domain (SPR & Docking confirmed); Protein stabilization	↓ Yoda1-induced Ca2+ influx; Severe mechanotransduction blockade	Speculative in IPF (In vitro & In vivo renal fibrosis model)	(63)
	Celastrol	CAND1 (Cys264), PCAF	Covalent conjugation (Michael addition); Direct non-covalent binding	↓ Myofibroblast transformation (FMT); ↓ NF-κB acetylation	Preclinical (In vitro & In vivo BLM IPF model)	(64)
	Eucalyptol	STAT6, p38 MAPK	Nuclear localization inhibition; Phosphorylation blockade	↓ IL-13-induced profibrotic macrophage polarization; ↓ TGF-β1 secretion	Preclinical (In vivo BLM IPF model)	(65)

### 4.1 Flavonoids and polyphenols: molecular precision and metabolic targeting

Flavonoids and polyphenols are extensively characterized macrophage modulators. Their pharmacological activity is determined by their core scaffold: the C2=C3 double bond provides the molecular planarity required for π-π stacking within kinase ATP-binding pockets, while specific B-ring hydroxylation patterns (particularly the catechol moiety) determine hydrogen-bonding affinity (59, 60).

Baicalein (5,6,7-Trihydroxyflavone): The Metabolic Reprogrammer. Baicalein is a therapeutic agent that targets multiple aspects of macrophage immunometabolism. A 2025 study using ABPP showed that baicalein acts as a targeted covalent inhibitor of glycolysis. Oxidation of its 5,6,7-trihydroxy A-ring forms an electrophilic quinone methide intermediate. This intermediate covalently binds the catalytic cysteine residue (Cys150) of GAPDH via Michael addition (47). This covalent modification suppresses glycolytic flux, inducing a metabolic shift toward OXPHOS and reversing the profibrotic Mo-AM phenotype (61). Additionally, scRNA-seq data indicate a dual-cellular targeting mechanism, where baicalein suppresses HIF-1α-driven glycolysis in both macrophages and adjacent fibroblasts, disrupting their metabolic crosstalk (61). Baicalein also competitively inhibits the KEAP1-NRF2 protein-protein interaction (62) and functions as an ATP-competitive inhibitor of the PI3K catalytic subunit, blocking fibrotic signaling networks (40).

Quercetin and Salvianolic Acid B: Pathway Modulators. Quercetin features a penta-hydroxy structure and a B-ring catechol. It modulates immune responses by suppressing follistatin-like 1 (FSTL1) expression, which blocks the NF-κB p65 nuclear translocation required for initial inflammatory signaling (63). Salvianolic Acid B (SAB), a polyphenol containing caffeic acid moieties, attenuates profibrotic macrophage activation by inhibiting the Mincle-Syk-PKCδ kinase cascade. SAB also exhibits anti-senescence properties. It inhibits the binding of the SP1 transcription factor to P21 and P16 promoters. This suppresses the SASP in both macrophages and AT2 cells, reducing paracrine recruitment signals within the fibrotic niche (64).

Curcumin and Resveratrol: Curcumin contains a bis-α,β-unsaturated β-diketone structure that activates PPARγ. This receptor subsequently heterodimerizes with RXR-α to upregulate cathepsins B and L, which restores proteolytic balance and promotes collagen degradation (65). Resveratrol functions as an allosteric activator of the SIRT1/SIRT3 network. This initiates a SIRT3/AMPK/autophagy feedback loop that alters macrophage bioenergetics and reduces oxidative stress-associated fibrosis (66).

### 4.2 Alkaloids: bioenergetic shifting and calcium channel blockade

Alkaloids contain nitrogenous heterocycles and primarily target bioenergetic sensors and ion channels in the fibrotic niche.

Berberine: The AMPK-Dependent Metabolic Shifter. Berberine is an isoquinoline alkaloid with a protoberberine tetracyclic scaffold and a quaternary nitrogen. This planar, positively charged structure facilitates mitochondrial accumulation. Berberine alters macrophage metabolism by acting as a direct AMPK activator. It inhibits mitochondrial complex I, increasing the AMP/ATP ratio, and directly binds AMPK regulatory subunits (67). This AMPK activation inhibits the mTORC1/HIF-1α axis, suppressing the transcription of glycolytic enzymes such as HK2 and LDHA. By reducing the glycolytic dependence of Mo-AMs, berberine induces a metabolic shift from glycolysis to OXPHOS, disrupting the metabolic basis of the profibrotic Mo-AM state (67).

Tetrandrine: Dampening Mechanosensitive Calcium Influx. Tetrandrine is a bisbenzylisoquinoline alkaloid that acts as an L-type calcium channel blocker. By reducing intracellular Ca^2+^ influx in macrophages, tetrandrine attenuates calcium-dependent signaling pathways involved in mechanosensitive responses. It also inhibits signal-induced degradation of IκBα, which prevents NF-κB nuclear translocation and dampens the STING-TBK1 inflammatory signaling axis, reducing profibrotic macrophage activation (67).

### 4.3 Terpenoids: direct mechanosensor inhibition and covalent conjugation

Terpenoids, composed of isoprene units, provide specific pharmacological interventions in fibrotic diseases, particularly by modulating mechanotransduction.

Paeoniflorin: The Direct Piezo1 Inhibitor. Paeoniflorin is a monoterpene glycoside identified as a direct inhibitor of the mechanosensitive ion channel Piezo1. With a rigid pinane skeleton linked to a glucose moiety, paeoniflorin binds to the Piezo1 pocket. This mechanism has been confirmed via Surface Plasmon Resonance (SPR) and molecular docking (55). By binding to Piezo1, paeoniflorin inhibits Yoda1-induced and stiffness-induced Ca2+ influx. This blockade prevents the subsequent stabilization of HIF-1α and the nuclear translocation of YAP/TAZ, reducing macrophage sensitivity to the 10–20 kPa stiffness of the surrounding ECM. By interrupting this Piezo1/Ca2+/HIF-1α mechanosensory loop, paeoniflorin attenuates fibrogenesis (55).

Celastrol: Covalent Targeting via Michael Addition. Celastrol is a pentacyclic triterpenoid containing a quinone methide moiety with an α,β-unsaturated carbonyl. This structure functions as a Michael acceptor, allowing for covalent modification of target proteins. ABPP data demonstrate that celastrol covalently conjugates to Cys264 of CAND1 (cullin-associated and neddylation-dissociated 1), increasing the activity of SCF ubiquitin ligases and inhibiting FMT (68). Additionally, it binds and suppresses PCAF (a histone acetyltransferase), preventing the acetylation-dependent activation of NF-κB (68).

**Figure 2:**
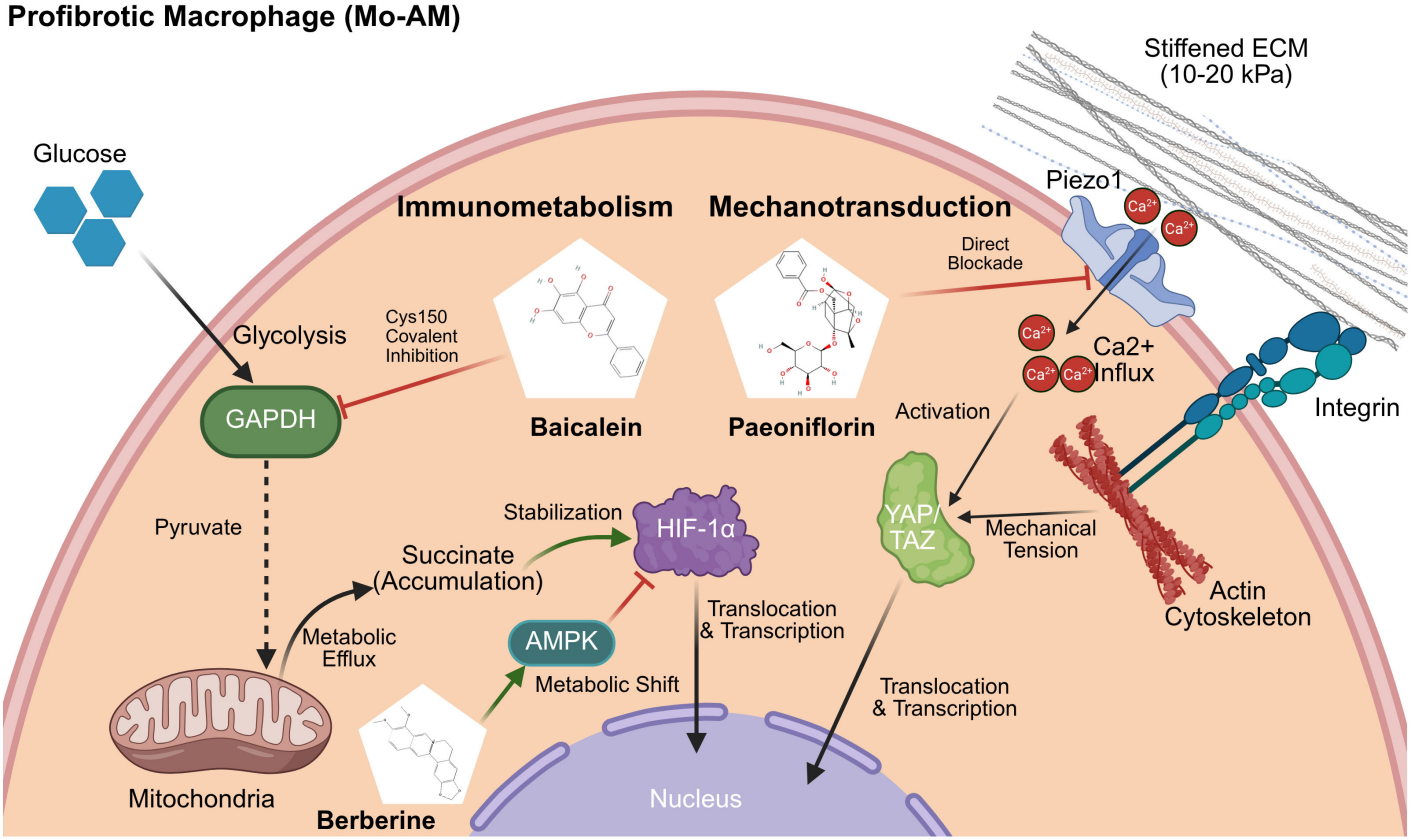
Intracellular rewiring of pro-fibrotic Mo-AMs and targeted interventions by natural monomers (Created with BioRender.com.). The pro-fibrotic polarization of Mo-AMs is governed by the intricate crosstalk between immunometabolism and mechanotransduction. (Left) In the immunometabolic axis, enhanced glycolysis catalyzed by GAPDH leads to mitochondrial accumulation and subsequent efflux of succinate. Elevated succinate stabilizes HIF-1α by inhibiting prolyl hydroxylases, promoting its nuclear translocation and the transcription of fibrotic genes. (Right) In the mechanotransduction axis, the stiffened ECM physically triggers the opening of Piezo1 channels, causing intracellular Ca^2+^ influx. Simultaneously, ECM stiffness engages integrins to induce actin cytoskeleton remodeling. These physical and chemical signals synergistically activate YAP/TAZ, driving their nuclear translocation. Natural compounds act as targeted pharmacological agents to dismantle this network: Baicalein covalently inhibits GAPDH at Cys150 to disrupt glycolysis; Paeoniflorin directly blocks the Piezo1 mechanosensor; and Berberine induces a metabolic shift via AMPK activation, thereby suppressing HIF-1α stabilization. (Note: The pharmacological mechanisms illustrated are predominantly based on preclinical *in vitro* assays and experimental non-IPF animal models. For example, the covalent inhibition of GAPDH at Cys150 by baicalein is extrapolated directly from an acute lung injury (ALI) model rather than an established IPF model. Therefore, these representations should be interpreted as proposed investigational pathways rather than clinically validated targets in human IPF.).

Eucalyptol and Asiatic Acid. Eucalyptol, featuring a bicyclic ether structure (1,8-cineole), blocks the phosphorylation of STAT6 and p38 MAPK. This kinase inhibition downregulates downstream transcription factors (KLF4, PPARγ), preventing IL-13-induced profibrotic macrophage polarization and reducing paracrine TGF-β1 secretion (69). The pentacyclic triterpenoid Asiatic acid acts as a competitive inhibitor of the Keap1-Nrf2 complex. Its hydroxyl groups enable binding to the Keap1 Kelch domain. This liberates Nrf2 to translocate to the nucleus, initiating an antioxidant response that mitigates ROS-associated fibrotic cascades (69). The comprehensive networks of these intracellular rewiring mechanisms and the targeted interventions by representative natural monomers are systematically illustrated in **Figure 2**.

### 4.4 Carbohydrate polymers: mitochondrial repair and dual antioxidant mechanisms

Beyond the aforementioned small molecules, naturally derived carbohydrate polymers such as chitosan oligosaccharides (COS) represent an emerging class of multi-target antifibrotic therapeutics. Recent studies have demonstrated that COS effectively combat pulmonary fibrosis by targeting mitochondrial dysfunction and oxidative stress within the fibrotic niche. Specifically, COS exert their protective effects through dual antioxidant mechanisms and mitochondrial repair pathways, which prevents fibrotic progression and restores cellular bioenergetics. These modes of action align closely with the concepts of immunometabolic reprogramming and microenvironmental modulation, further enriching the multi-target therapeutic landscape of natural products against IPF (70, 71).

## 5 Traditional formulas: holistic and synergistic reprogramming of the niche

Although targeting isolated natural monomers provides mechanistic insights, the redundancy of the fibrotic niche often limits the efficacy of single-target interventions. TCM formulas address this limitation through multi-component and multi-target interactions. These formulas inhibit profibrotic macrophage activation and modulate the overall fibrotic microenvironment. The regulatory networks, macrophage-modulating outcomes, and validation strategies of these representative TCM formulas are summarized in **Table 2**.

**Table 2:** Modulating the fibrotic niche by traditional Chinese medicine formulas: network mechanisms and evidence stratification.

TCM formula	Key active components/Q-markers	Primary regulated pathways	Macrophage & microenvironmental outcomes	Validation strategy & evidence level	Ref.
Renshen Pingfei Formula (RSPF)	78 components identified (incl. Kaempferol, Luteolin)	TGF-β/Smad, AMPK/PPAR-γ	↓ Profibrotic markers (Arg-1, CD206) in alveolar/interstitial niches; ↓ ECM deposition	Preclinical (In vivo) & Clinical plasma metabolomics	(66, 72)
Maimendong Decoction (MMDD)	Multi-component matrix	PI3K/Akt/FOXO3a	Attenuates profibrotic Mo-AM state; ↓ Macrophage secretion of TGF-β1 & PDGF-RB	Preclinical (In vitro/In vivo BLM IPF model)	(55)
Fuzheng Huayu Recipe (FZHYR)	Multi-component matrix	METTL3-mediated m6A modification (Epigenetic)	↓ OXPHOS in pathogenic macrophages; ↓ Profibrotic Mo-AM activation	Preclinical (In vitro mechanistic validation)	(68)
Buyang Huanwu Decoction (BYHWD)	Blood stasis-resolving components	HMGB1/NF-κB	Attenuates localized hypoxia & platelet activation; Shifts niche to pro-resolving state	Preclinical (In vivo BLM IPF model)	(69)
Qingfeiyin Decoction (QFY)	Baicalin, Oroxylin A, Geniposide	PI3K (p110α and p110γ subunits)	↓ Profibrotic macrophage polarization; ↓ FMT and EMT	Computational (Molecular dynamics) & In vitro	(19)
Jinshui Huanxian Granules (JSHX)	266 components (incl. Flavonoids, Saponins, Alkaloids)	MAPK cascade, Oxidative stress pathways	↓ α-SMA and fibronectin; Modulates immune cell migration	Analytical (MS fingerprinting) & Computational	(73)
Shuangshen Pingfei Formula (SSPF)	5 validated Q-markers (e.g., Salvianolic acid B, Mangiferin)	CCL2/CCR2 recruitment axis	↓ Macrophage recruitment to the lung; Downregulates specific chemokine receptors	Preclinical (In vivo pharmacokinetics)	(74)
Jinbei Oral Liquid (JBOL)	Astragalus membranaceus, Codonopsis pilosula	Multi-pathway synergistic network	Statistically significant reduction in total lung capacity (TLC) decline	Clinical (Double-blind, multicenter RCT)	(70)

### 5.1 Synergistic effects and holistic niche reprogramming

Recent studies indicate that TCM formulas function as integrated pharmacological networks. Instead of inhibiting a single kinase, these formulas suppress both macrophage polarization and downstream fibroblast activation by modulating key regulatory pathways (PI3K/AKT, TGF-β/Smad, HIF-1α, and AMPK/PPAR-γ) (19, 72).

Dual Suppression of Profibrotic Macrophage Activation and Fibrotic Signaling: The Renshen Pingfei Formula (RSPF) demonstrates this multi-target effect. RSPF downregulates profibrotic macrophage markers (Arg-1, CD206) in both the alveolar and interstitial compartments. Concurrently, it suppresses TGF-β and Smad2/3 phosphorylation to reduce ECM deposition (19). Similarly, the Maimendong Decoction (MMDD) inhibits the PI3K/Akt/FOXO3a pathway, reducing the secretion of profibrotic mediators, such as TGF-β1 and PDGF-RB, from macrophages (75). Additionally, the Fuzheng Huayu Recipe (FZHYR) modulates epigenetic regulation by inhibiting profibrotic Mo-AM activation via METTL3-mediated m6A modification of NDUFA2, which reduces OXPHOS in pathogenic macrophages (76).

Microenvironmental Modulation: In addition to targeting macrophages, blood stasis-resolving formulas modulate the fibrotic niche. The Buyang Huanwu Decoction (BYHWD) attenuates localized hypoxia, platelet activation, and inflammation via HMGB1/NF-κB pathway regulation (73). By modulating immune infiltration, ECM remodeling, and cellular metabolism, these formulas show clinical efficacy. Recent randomized controlled trials (RCTs) demonstrate statistically significant reductions in total lung capacity decline in patients treated with specific formulas compared to placebo (74).

### 5.2 Modern bioinformatics and network pharmacology

The integration of bioinformatics and network pharmacology has clarified the mechanisms of TCM formulas. This in silico to *in vitro*/*in vivo* workflow identifies the active components and core target networks of complex formulas (77).

Standardized Computational Workflows: Researchers integrate databases (e.g., TCMSP, GeneCards) with clinical transcriptomics (GEO datasets) to identify differentially expressed genes (DEGs) in IPF patients (72, 77). By constructing protein-protein interaction (PPI) networks using STRING and topological algorithms, studies identify hub nodes (e.g., AKT1, MAPK3, VEGFA) that mediate macrophage-driven fibrogenesis (72).

Molecular Docking and Dynamics Validation: To validate these networks, studies use molecular docking (AutoDock Vina) and molecular dynamics simulations (100–200 ns). For example, in the analysis of Qingfeiyin Decoction (QFY), molecular simulations showed that its bioactive constituents, baicalin and oroxylin A, exhibit stable binding affinities to the PI3K p110α and p110γ subunits (78).

Integration with Clinical Metabolomics: Recent approaches integrate untargeted metabolomics with network predictions. By employing UPLC-MS/MS to analyze clinical plasma metabolomics pre- and post-treatment, researchers correlate theoretical bioinformatic targets with actual metabolic shifts in patients (79).

### 5.3 Quality control requirement and chemical fingerprinting

Standardized quality control is necessary to address reproducibility issues associated with multi-component formulas in pharmacological research. Current methodologies require the use of Quality Markers (Q-markers) and chemical fingerprinting to ensure batch-to-batch consistency and link biological effects to specific active components (80, 81).

High-Resolution Chromatographic Profiling: Analytical platforms, such as UPLC-Orbitrap Fusion MS and UHPLC/Q-TOF-MS, improve TCM quality control. For example, the chemical profiling of Jinshui Huanxian Granules (JSHX) identified 266 distinct components and mapped 90 validated components to 172 fibrosis-related targets (82). This high-resolution mass spectrometry enables MS fragmentation pattern analysis and automatic database screening, providing empirical chemical evidence for theoretical predictions.

The Q-Marker Framework: The establishment of validated Q-markers integrates phytochemical analysis, pharmacokinetics, and pharmacodynamics. In the evaluation of the Shuangshen Pingfei Formula, researchers used principal component analysis (PCA) across multiple batches to verify baseline consistency. Concurrently, pharmacokinetic studies (UPLC-MS/MS) confirmed that specific marker compounds (e.g., salvianolic acid B, mangiferin) reached systemic circulation and downregulated the CCL2/CCR2 macrophage recruitment axis in the lung (83).

Linking pharmacological efficacy to chemical fingerprints and molecular docking data improves the reliability of these studies. This quality control ensures that the macrophage-modulating effects of TCM formulas are reproducible and clinically applicable.

## 6 Challenges and future perspectives

While preclinical studies demonstrate the antifibrotic effects of natural therapeutics, translating these findings into approved clinical interventions faces pharmacokinetic, methodological, and clinical challenges. As highlighted by recent literature on emerging therapeutic strategies for IPF (8, 9), overcoming these translational hurdles is critical. Specifically for natural product-based antifibrotic strategies (17), the development of IPF therapies requires integrating materials science, empirical multi-omics, and rigorous clinical trial designs. This integration is necessary to evaluate the efficacy of natural monomers and TCM formulas.

### 6.1 Pharmacokinetic bottlenecks and advanced nano-delivery systems

Natural products and TCM compounds have physicochemical limitations that restrict their therapeutic index. Natural monomers frequently exhibit poor aqueous solubility, extensive first-pass metabolism, and rapid hepatic clearance. These factors lead to insufficient drug accumulation in the lungs (17). Additionally, the fibrotic lung acts as a physical barrier. Excessive collagen deposition forms a dense ECM that limits drug penetration to AT2 cells and myofibroblasts. For TCM formulas, conventional systemic administration does not maintain the synchronized pharmacokinetic profiles of multiple active ingredients. This results in spatial and temporal separation, which reduces their synergistic effects (84).

To address these barriers, researchers are developing macrophage-targeted nano-delivery systems. For monomeric compounds, functionalized nanocarriers, such as M2pep-modified metal-organic frameworks and mannosylated albumin nanoparticles, target the upregulated mannose receptors (CD206) on profibrotic Mo-AMs (85). These platforms facilitate intracellular drug delivery. This approach reduces pathogenic Mo-AM populations while preserving homeostatic TR-AMs.

For TCM formulas, nano-co-delivery systems integrate multi-component formulations with modern drug delivery. For example, macrophage membrane-camouflaged biomimetic nanoparticles (e.g., PCMΦ@PPT) can be co-loaded with multiple active components, such as tetrandrine and pirfenidone. These inhalable nanocarriers are coated with collagenase and collagen-binding peptides. They degrade the fibrotic ECM to facilitate tissue penetration, evade immune clearance, and extend pulmonary retention up to 72 hours (86, 87). By delivering multiple compounds to the fibrotic niche at specific ratios, these platforms maintain the network pharmacology of TCM and increase localized bioavailability.

### 6.2 Bridging the translational gap via empirical multi-omics

TCM pharmacological research currently faces methodological limitations regarding component identification. Many network pharmacology studies rely on public computational databases (e.g., TCMSP) to predict active ingredients. This approach produces predictions that often do not reflect the actual chemical composition, tissue-specific bioavailability, or physiological interactions within the pulmonary microenvironment (88).

Future research requires a shift from theoretical database predictions to empirical validation using raw metabolomics data. By applying serum pharmacochemistry and high-resolution untargeted metabolomics (such as UHPLC-HRMS), researchers can identify the bioactive constituents that enter the systemic circulation and accumulate in lung tissue (79, 89). Compounds verified through experimental mass spectrometry data provide a more accurate representation of a formula’s material basis than theoretical algorithms.

**Figure 3:**
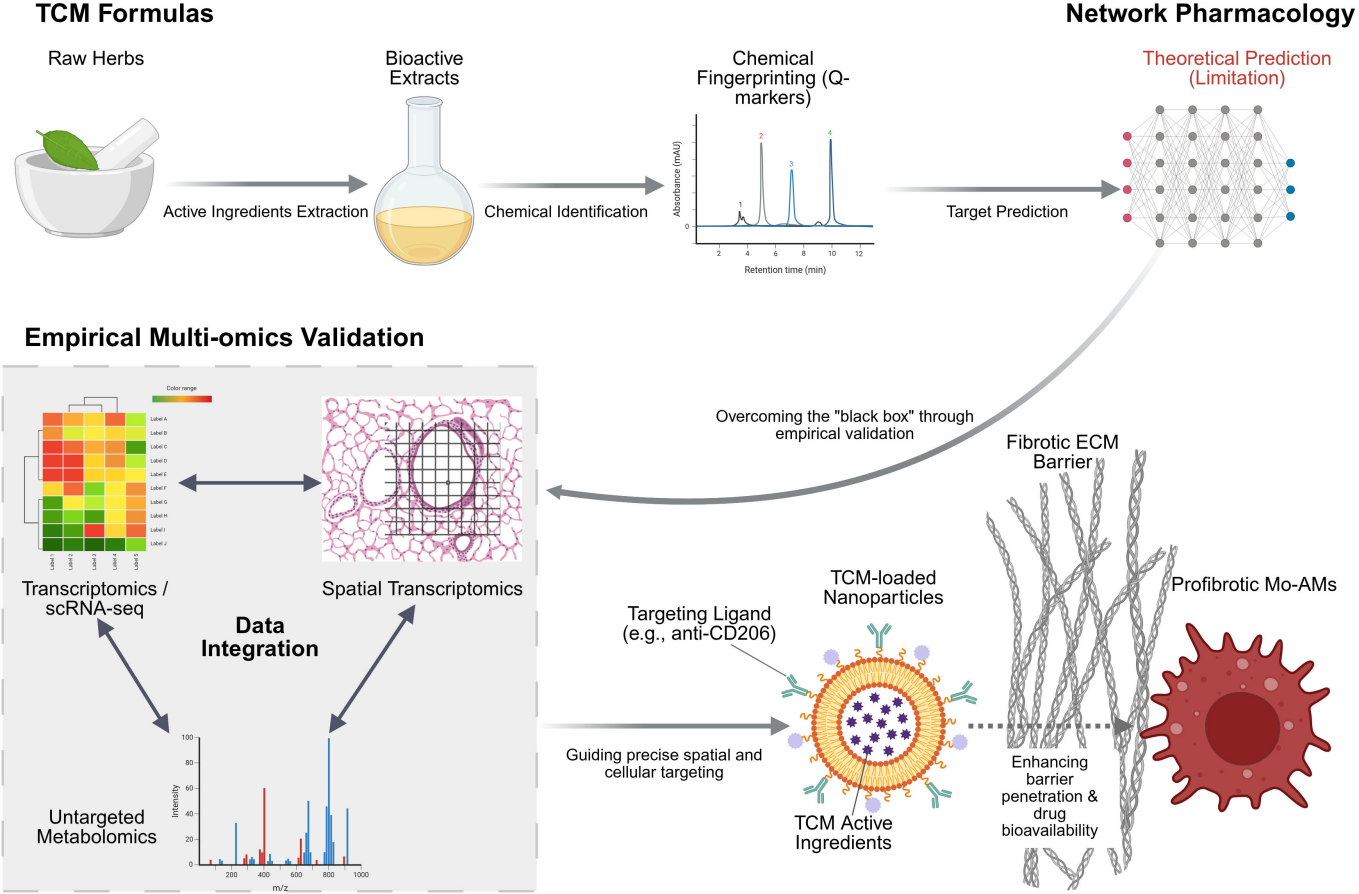
Future perspectives: integrating multi-omics and targeted nano-delivery to modernize TCM therapies for IPF (Created with BioRender.com.). To overcome the limitations of theoretical network pharmacology, the modernization of TCM requires a multi-dimensional empirical approach. Bioactive extracts from TCM formulas are chemically identified (e.g., via mass spectrometry) to establish Q-markers for target prediction. Crucially, these theoretical networks must be validated through integrated multi-omics, incorporating transcriptomics (scRNA-seq), spatial transcriptomics, and untargeted metabolomics to map the precise spatiotemporal alterations within the fibrotic niche. To translate these empirical findings into clinical efficacy, advanced nano-delivery systems are proposed. By encapsulating TCM active ingredients into functionalized nanoparticles engineered with targeting ligands (e.g., anti-CD206), these systems can effectively penetrate the dense fibrotic ECM barrier, ensuring precise spatial localization and enhanced drug bioavailability directly within pro-fibrotic Mo-AMs. (This figure illustrates a proposed translational roadmap. The nano-delivery systems and multi-omics integration strategies reflect current preclinical methodologies aiming for future clinical application.).

Evaluating the multi-target effects of TCM formulas also requires spatial mapping. While scRNA-seq identifies cellular heterogeneity, it removes the architectural context of the tissue. Therefore, spatial transcriptomics is necessary (90). By preserving the spatial distribution of gene expression, spatial transcriptomics demonstrates how a multi-component formula affects different cell types simultaneously. For example, it can show the concurrent suppression of SPP1-high macrophages in the airway, the inhibition of FMT in fibroblastic foci, and the restoration of AT2 cells (91). Integrating clinical metabolomics with spatial transcriptomics provides comprehensive data to validate TCM network pharmacology. This translational roadmap, integrating empirical multi-omics validation with targeted nano-delivery systems for TCM formulas, is proposed in **Figure 3**.

### 6.3 Rigorous clinical translation and quality standards

The clinical translation of natural therapeutics for IPF requires high-quality, reproducible evidence. Previous RCTs evaluating TCM interventions have shown methodological limitations. These include inadequate allocation concealment, insufficient blinding, and reliance on subjective syndrome-scoring endpoints (92). The personalized nature of TCM treatment protocols often conflicts with the standardized requirements of modern RCTs, complicating the evaluation process (93).

Future clinical trials must implement strict methodological standards. First, botanical formulas require chemical standardization using the Q-marker framework. Before clinical evaluation, the metabolomic signatures of the formula must be analyzed via LC-MS/MS fingerprinting. This ensures batch-to-batch consistency and confirms that the clinical intervention matches the chemical profile evaluated in preclinical studies (94).

Second, clinical trial designs must adhere to the CONSORT-CHM extension guidelines and utilize objective primary endpoints (95). According to global respiratory society guidelines, the absolute decline in FVC over 52 weeks is the standard measure of therapeutic efficacy, supported by metrics such as DLCO trajectory and time to acute exacerbation (96). Combining chemical quality control with objective physiological endpoints is necessary to validate natural therapeutics as viable treatments for pulmonary fibrosis.

### 6.4 Safety considerations for natural therapeutics

While natural therapeutics are often perceived by the general public as inherently safe, this assumption is a dangerous misconception. Several bioactive natural compounds exhibit significant dose-dependent toxicities and narrow therapeutic windows, which heavily limit their direct clinical translation. For instance, celastrol, despite its potent anti-fibrotic efficacy via covalent protein modifications, is associated with well-documented hepatotoxicity and systemic adverse effects (97). Similarly, the alkaloid tetrandrine can induce liver damage and potential immunosuppression at elevated doses, often mediated through the disruption of redox homeostasis and specific enzyme inhibition (98). Furthermore, compounds like glycyrrhizic acid, widely utilized as a harmonizing agent in many TCM formulas, can cause pseudoaldosteronism, leading to severe hypertension and hypokalemia due to the inhibition of 11β-hydroxysteroid dehydrogenase type 2 in the kidneys (99). Therefore, future clinical applications require rigorous and systematic toxicity profiling. Developing structural analogs to reduce off-target effects and utilizing targeted nano-delivery systems (as discussed in Section 6.1) are essential strategies to maximize localized efficacy while minimizing systemic toxicity.

## 7 Conclusion

In conclusion, IPF progression is driven by a self-perpetuating fibrotic niche. Within this microenvironment, monocyte-derived alveolar macrophages regulate disease activity through immunometabolic reprogramming and mechanotransduction. The spatiotemporal and physical complexity of this microenvironment limits the efficacy of conventional, single-target antifibrotic therapies. Consequently, natural therapeutics represent a viable investigational approach.

A dual-strategy approach can effectively modulate the fibrotic microenvironment. Natural monomers, characterized by structural specificity, directly inhibit specific profibrotic pathways. Examples include the covalent inhibition of glycolytic GAPDH by baicalein and the direct blockade of the Piezo1 mechanosensor by paeoniflorin. Concurrently, quality-controlled traditional formulas provide synergistic effects. These formulas modulate multi-cellular interactions, attenuate oxidative stress, and restore microenvironmental homeostasis in experimental models.

The clinical translation of these therapeutics requires a shift from theoretical, database-driven methodologies to empirical validation. Future research should utilize empirical multi-omics. Integrating raw metabolomics identifies *in vivo* active constituents, while spatial transcriptomics maps regulatory networks across the fibrotic niche. These approaches systematically validate the molecular basis of TCM. Combined with macrophage-targeted nano-delivery systems and rigorous chemical fingerprinting in clinical trials, natural therapeutics can transition from empirical remedies to evidence-based therapeutic options for the treatment of IPF.
